# Assessing prognostic value of early tumor shrinkage and depth of response in first-line therapy for patients with advanced unresectable pancreatic cancer

**DOI:** 10.1186/s12876-021-01870-x

**Published:** 2021-07-15

**Authors:** Xiaojuan Yang, Xinghong Xian, Yongsheng Wang, Meng Qiu

**Affiliations:** 1 Division of Thoracic Oncology, Cancer Center, West China Hospital, Sichuan University, Chengdu, China; 2 Division of Abdominal Oncology, Cancer Center, West China Hospital, Sichuan University, No. 37 Guo Xue Xiang, Chengdu, 610041 China

**Keywords:** ETS, DpR, Recurrence, Metastasis, Pancreatic cancer, Prognosis

## Abstract

**Background:**

The prognostic potential of early tumor shrinkage (ETS) and depth of response (DpR) in pancreatic cancer (PC) is unclear. Here, we recruited 90 patients with recurrent and metastatic PC (RMPC) who had received chemotherapy as first-line therapy to assess the prognostic potential of these markers.

**Methods:**

ETS is characterized as a ≥ 20% depletion in the sum-of-the-longest-diameters (SLD) of measurable tumor lesions at 6–12 weeks than the baseline. DpR is the maximum shrinkage (%) from the baseline to nadir. We evaluated corrections in ETS and DpR with survival.

**Results:**

Of the 63 patients in which ETS assessment was possible, 21 (33.3%) achieved ETS. We found a significant association between the incidence of ETS and an improved rate of progression-free survival (PFS; 6.5 vs. 2.2 months; *p* < 0.001) and overall survival (OS; 12.1 vs. 6.0 months; *p* = 0.014). The median value of DpR was − 23.66%. DpR was also related to improved PFS (9.3 vs. 3.1 months; *p* < 0.001) and OS (18.2 vs. 7.3 months; *p* < 0.001). Patients who had distant metastasis, not local recurrence, with ETS showed markedly better outcomes. In a multivariate model, both ETS and DpR were independent predictors of OS in the whole population.

**Conclusions:**

ETS and DpR may predict favorable outcomes for RMPC patients who had received chemotherapy as first-line therapy, independent of the agents used. Further studies on the exploratory analyses of the optimum ETS cut-off value in recurrent PC patients to predict favorable clinical outcomes are required.

**Supplementary Information:**

The online version contains supplementary material available at 10.1186/s12876-021-01870-x.

## Background

The 5-year survival rate of pancreatic cancer (PC), a fatal disease, is 9% for all stages combined, and it ranks seventh in terms of global mortality due to cancer [1, 2]. Despite early diagnosis, it has a post-surgery 5-year survival rate < 20% and approximately 80% of the cases are known to relapse within 2 years [3–5]. Additionally, patients with metastasized PC have a 5-year survival rate of 3% [5].

Recurrent and metastatic pancreatic cancer (RMPC) remains an incurable disease. Currently, the systemic treatment options involve gemcitabine monotherapy or combined chemotherapy, including gemcitabine-based regimens and fluorouracil (5FU)-based regimens as the first-line therapy [6, 7]. Despite the availability of various types of treatment regimens for advanced PC, there is a lack of factors that define the precocity or depth of tumor regression and can reliably anticipate prognosis as the first-line of therapy.

Early tumor shrinkage (ETS) implies a ≥ 20% depletion in tumor burden measured after treatment initiation compared with that evaluated at the baseline [8]. Depth of response (DpR) is classified as the largest decrease (%) in the tumor size, calculated based on the reconstructed volume or longest diameters at the nadir than the baseline [9]. The post-hoc analysis of three randomized trials (OPUS, CRYSTAL, and FIRE-1) showed that ETS within 7–8 weeks post-treatment initiation was significantly related to a longer overall survival (OS) and progression-free survival (PFS) [9, 10]. Additionally, the post-hoc analyses of TRIBE, PEAK, and FIRE-3 trials demonstrated that DpR > median value was linked to a longer PFS, post-progression survival (PPS), and OS [11–13]. Thus, these studies confirmed that both ETS and DpR were related to a good prognosis in cases of metastatic colorectal cancer (mCRC), irrespective of the first-line systematic therapy received.

However, there is limited information available on the function of DpR and ETS and in the outcome prediction of RMPC. The first investigational study that examined ETS as a potentially favorable outcome predictor involved 59 subjects with advanced PC who were being treated with FOLFIRINOX (5-fluorouracil, irinotecan, and oxaliplatin) [14]. It reported that patients who achieved an ETS had a median value of OS and PFS as 24.0 months and 9.0 months, respectively, compared with 9.1 and 4.2 months for the non-ETS patients. The ETS was statistically significantly with PFS (*p* = 0.020) in multivariate analysis but was not statistical significance with OS (*p* = 0.065) [14]. Recently, a retrospective study conducted by Caterina V et al. on 138 patients with metastatic PC receiving either FOLFOXIRI (5-fluorouracil, irinotecan, and oxaliplatin) or Gemcitabine plus Nab-paclitaxel. The results showed that DpR and ETS were strongly related to OS and PFS in PC patients treated with FOLFOXIRI; however, no statistical correlation was observed in the Gemcitabine plus Nab-paclitaxel cohort [15]. Therefore, previous studies reported a conflict between the association of ETS and OS, indicating the need for further studies. Additionally, the prognostic potential of DpR and ETS in advanced PC patients who received chemotherapy as the first-line therapy is not completely understood, and thus, it is unclear whether the prognostic potential is independent of treatment administration.

Here, we examined the prognostic potential of ETS and DpR in RMPC patients who received chemotherapy as the first-line therapy, irrespective of their treatment regimens.

## Methods

### Patients

**Fig. 1:**
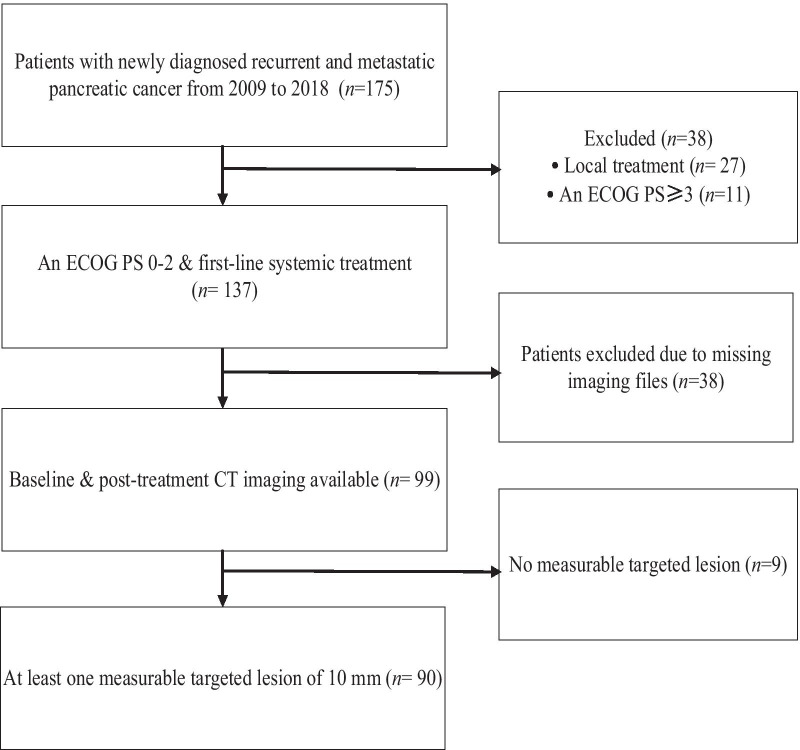
CONSORT flow chart for patient selection

We reviewed RMPC patients at the West China Hospital of Sichuan University database between 2009 and 2018 (Fig. 1). The enrolled patients were ≥ 18 years, had an Eastern Cooperative Oncology Group (ECOG) performance status (PS) of 0, 1, or 2, and had histopathologically confirmed recurrent and unresectable or metastatic PC. According to the Response Evaluation Criteria in Solid Tumors (RECIST), version 1.1 [16], eligible patients should have at least one 10 mm measurable targeted lesion, had at least one first-line systemic treatment at our institution, and had done their tumor assessments by Computed Tomography (CT) at least once. Cases without available imaging within 28 days before treatment initiation and lacking complete clinicopathologic and follow-up data were excluded. Patients who had a history of another major cancer, endocrine pancreatic carcinoma, and were pregnant or breastfeeding were also excluded.

### Evaluation of ETS and DpR

The investigators used the RECIST version 1.1 to assess the tumor responses [16]. The sum-of-the-longest-diameters (SLD) of RECIST tumor lesions at baseline and after treatment initiation were analyzed. ETS was classified as a ≥ 20% depletion in the SLD at 6–12 weeks after treatment initiation [17]; non-ETS included a minor shrinkage (a decrease by 0 to 19%), tumor growth, and new metastatic lesions [8]. DpR was characterized as the maximal decrease (%) from the baseline to nadir, without the appearance of novel lesions or the growth of non-target lesions [12]. DpR = [(SLD at nadir)—(SLD at baseline)] /(SLD at baseline) × 100% [15]. We evaluated ETS as both a continuous and a binary (≥ 20% vs. < 20%) variable. Also, DpR was treated either as continuous, or as an ordinal (with four levels based on quintile distribution), or as a binary (≥ median vs. < median) variable. The DpR quartiles were classified as follows: quartile I: − 100% to − 37.98%; quartile II: − 37.98% to − 23.66%; quartile III: − 23.66% to + 15.28%; quartile IV: + 15.28% to + 67.89% (“+”, i.e. “increase of tumor,” “–”, i.e. “tumor shrinkage”) [12, 15].

### Study endpoints

The co-primary endpoints included the relationship between ETS/DpR and PFS/OS among all the participants. We defined median OS as the duration between treatment initiation (day 1) to the first documented death, whatever the cause and median PFS was defined as time from the first day of treatment to the date of the first documented tumor growth or death, whatever the cause. We censored patients who were alive and who had no disease progression at their last follow-up visit or at the date of their last radiologic assessment. Additionally, those patients were also censored whose survival time, including PFS and OS, were unavailable.

### Statistical analysis

We used Kaplan–Meier curves to estimate the link between ETS/DpR with survival, including PFS/OS, and compared it with the log-rank test (two-sided). For OS, each factor was initially assessed for their prognostic effect using univariate Cox proportional hazards regression analysis (enter method) [17]. Variables with *p* < 0.2 in the univariate analyses were selected as explanatory determinants for a stepwise multivariate Cox proportional hazards regression model. Nominal variables were presented as percentages. The Cox regression model was used to calculate the hazard ratios (HRs) and 95% confidence intervals (CIs). A *p-*value < 0.05 for a two-sided test was regarded as statistically significant. All statistical analyses were done using IBM SPSS v25.0 (SPSS Inc., USA) and GraphPad Prism v8.0.2 (*GraphPad* Software, Inc., USA.).

## Results

### Patient characteristics at baseline

One hundred seventy-five patients were diagnosed with RMPC between 2009 and 2018, of which 90 were included based on the study criteria (Fig. 1). Table 1 lists the baseline characteristics of the study population.

**Table 1:** Baseline demographic and clinical characteristics

Characteristics	Patients *n* = 90	
	*n*	%
Age, years		
Median	59	–
Range	38–89	–
< 65	63	70
≥ 65	27	30
Sex		
Male	57	63.3
Female	33	36.7
ECOG performance status		
0	44	48.9
1	27	30.0
2	19	21.1
Histology		
Adenocarcinoma	89	98.9
Adenosquamous carcinoma	1	1.1
Tumor site		
Head-uncinate process	41	45.6
Body-tail	47	52.2
Multifocal	2	2.2
Synchronous disease		
Yes	47	64.4
No	26	35.6
No. of metastatic sites		
1–2	61	83.6
3–4	12	16.4
Localization of metastasis		
Liver	61	67.8
Peritoneal	13	14.4
Lung	6	6.7
Bones	2	2.2
Local recurrence (including regional lymph node metastases)	17	18.9
Previous treatments		
Radical surgery	43	47.8
Adjuvant chemotherapy	24	26.7
Ca19.9 (KU/L)		
≤ ULN	15	16.7
> ULN	75	83.3
Biliary stent		
Yes	3	3.3
No	87	96.7
HDL-C (mmol/L)		
> 0.9	77	85.6
≤ 0.9	13	14.4

*Ca19.9* carbohydrate antigen 19–9, *DpR* depth of response, *ECOG* Eastern Cooperative Oncology Group, *ETS* early tumour shrinkage, *IPMN* intraductal papillary mucinous neoplasm, *ULN* upper limit of normal, *HDL-C* High-density lipoprotein cholesterol

### Treatment regimens and efficacy

The present analysis included 90 patients with different first-line treatment regimens. Specifically, 41 patients (46%) were treated with gemcitabine plus S-1 (GS); 14 patients (16%) received FOLFIRINOX. Approximately 8 (9%) and 6 (7%) patients were administered GEMOX (Gemcitabine and Oxaliplatin) and Gemcitabine plus Nab-paclitaxel, respectively. Additionally, there were 11 less frequently chosen regimens (data are given in Additional file 1: Table S1). Furthermore, one patient was given three cycles of GS and three cycles of Gemcitabine plus Nab-paclitaxel.

Each patient received the assigned therapy for four cycles in the median (1–10), with a median follow-up of 12.2 months. Amongst the study participants, the median OS and PFS were 8.8 and 4.4 months, respectively. The disease control rate (DCR) was 52.2% (47 patients), and the objective response rate (ORR) was 28.9% (26 patients, 22 achieving partial responses and 4 complete responses), and the based on RECIST v1.1 criterion. The death of 66 patients (73.3%) was recorded during the analysis. Meanwhile, the disease progressed in 89 patients (98.9%), of which 44 (49.4%) received second-line therapy. There were 18 different types of regimens for second-line therapy, of which the most frequently chosen was FOLFIRINOX (31.8%). Three patients (3.3%) had a biliary stent who had a poor prognosis with a median OS and PFS of 6.6 months and 1.4 months, respectively.

### ETS and DpR

Of the 90 patients, 63 were evaluated for the ETS. The development of new metastatic lesions was observed in 17 patients at 6–12 weeks after treatment initiation. Among the remaining 46 patients, the median reduction of the SLD from baseline was − 10.22%, and 21 patients (33.3%) achieved ETS.

Among 62 assessable patients, the median DpR was − 23.66% (from − 100 to + 67.89). Table 2 reports the quartile distribution of DpR, considering DpR as a continuous variable.

**Table 2:** Distribution of ETS and DpR

ETS and DpR cut-offs	Patients (*n*)	%
ETS	63	–
≥ 20%	21	33.3
< 20%	42	66.7
DpR	62	–
I quartile (− 100% to − 37.98%)	16	25.4
II quartile (− 37.98% to − 23.66%)	15	23.8
III quartile (− 23.66% to + 15.28%)	15	23.8
IV quartile (+ 15.28% to + 67.89%)	16	25.4
< Median	31	50
≧ Median	31	50

*DpR* depth of response, *ETS* early tumour shrinkage

### Prognostic factors univariate analyses

Amongst the unselected patients, both ETS (achieving ETS and ETS as a continuous variable) and DpR (DpR ≥ median and DpR as as a continuous variable) had greater univariate analyses values, which were significantly related to better PFS and OS (all *p* < 0.05) (Table 3).

**Table 3:** Association between clinicopathological features and survival parameters

Clinicopathological features	PFS		OS	
	HR (95% CI)	*P-*value	HR (95% CI)	*P-*value
Age (≥ 65 years)	0.741 (0.460–1.192)	0.216	0.653 (0.377–1.131)	0.128
ECOG PS (2)	9.725 (4.78–19.785)	** < 0.001**	5.200 (2.711–9.974)	** < 0.001**
Gender (female)	1.037 (0.665–1.618)	0.872	1.192 (0.708–2.006)	0.508
Sites of metastases (liver)	1.425 (0.909–2.235)	0.123	1.948 (1.088–3.487)	**0.025**
Sites of metastases (lung)	1.105 (0.478–2.555)	0.815	0.557 (0.169–1.839)	0.337
Sites of metastases (peritoneum)	1.646 (0.900–3.011)	0.106	1.002 (0.508–1.976)	0.995
Local recurrence (including regional lymph node metastases)	1.237 (0.636–2.407)	0.531	1.140 (0.486–2.673)	0.763
Number of metastatic sites (≥ 3)	2.239 (1.197–4.186)	**0.012**	2.090 (1.027–4.252)	**0.042**
Synchronous disease (no)	0.860 (0.533–1.390)	0.539	0.815 (0.460–1.445)	0.484
Tumor site (body-tail)	1.057 (0.741–1.508)	0.759	0.797 (0.533–1.192)	0.270
Surgery for primary tumor (yes)	0.782 (0.513–1.194)	0.255	0.815 (0.492–1.349)	0.425
CA-199 (KU/L) (> ULN)	1.532 (0.887–2.645)	0.126	1.415 (0.737–2.716)	0.297
HDL-C (mmol/L) (> 0.9)	1.560 (0.819–2.971)	0.177	1.104 (0.570–2.138)	0.770
ETS (≥ 20%)	0.523 (0.315–0.868)	** < 0.001**	0.699 (0.387–1.263)	**0.014**
ETS (as a continuous variable)	1.015 (1.010–1.020)	** < 0.001**	1.011 (1.006–1.017)	** < 0.001**
DpR (≥ median)	0.163 (0.092–0.290)	** < 0.001**	0.252 (0.132–0.480)	** < 0.001**
DpR (as a continuous variable)	1.035 (1.025–1.046)	** < 0.001**	1.022 (1.013–1.031)	** < 0.001**

Bold values indicate statistical significance at* P* < 0.05

*Ca19.9* carbohydrate antigen 19–9, *CI* confidence interval, *DpR* depth of response, *ECOG* Eastern Cooperative Oncology Group, *ETS* early tumour shrinkage, *ULN* upper limit of normal, *HDL-C* High-density lipoprotein cholesterol

For the univariate analyses, variables that were significantly correlated to unfavorable PFS included ECOG PS 2 (median PFS 1.8 vs. 5.3 months, *p* < 0.001) and the total metastatic sites ≥ 3 (median PFS 2.3 vs. 5.0 months, *p* = 0.012), whereas ECOG PS 2 (median OS 4.5 vs. 11.1 months, *p* < 0.001), total metastatic sites ≥ 3 (median OS 7.7 vs. 9.5 months, *p* = 0.042), and liver metastasis (median OS 4.0 vs. 10.9 months, *p* = 0.025) were considerably related to inferior OS (Table 3).

### ETS and its stratification analysis: correlation with survival

**Fig. 2:**
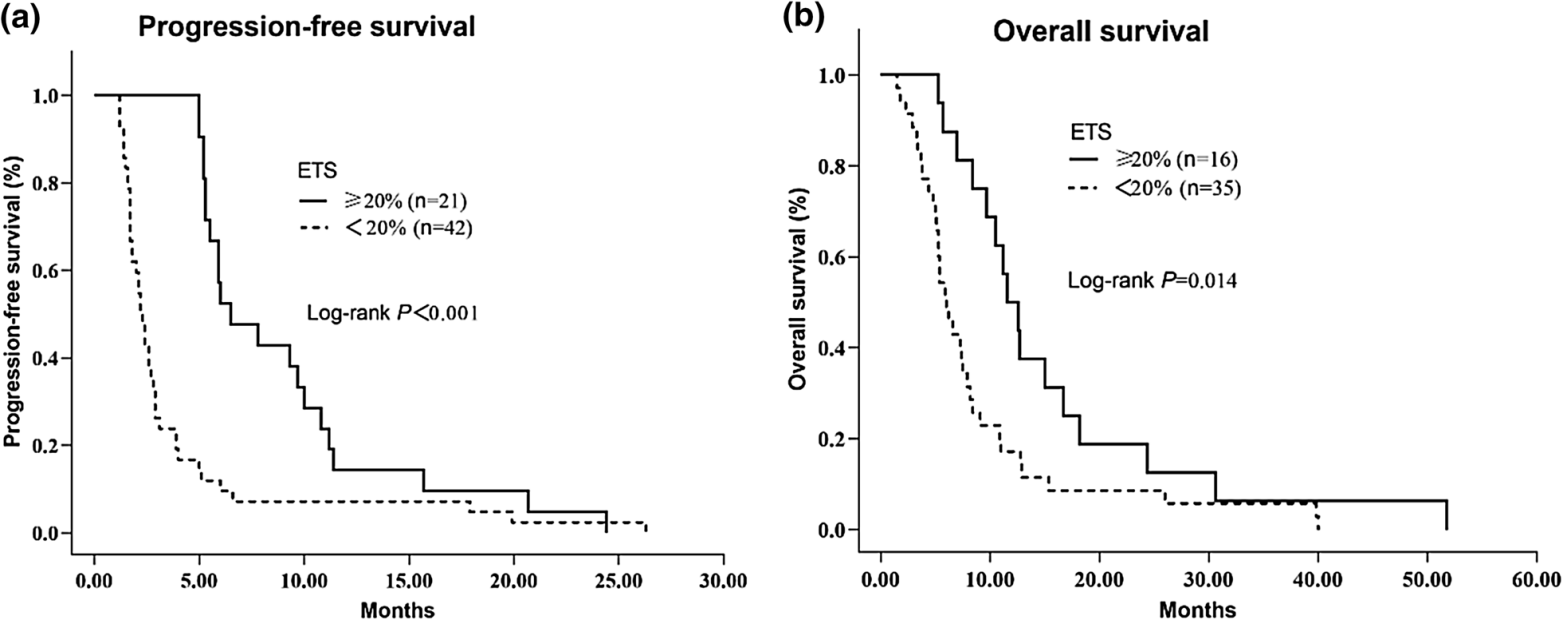
**a** Kaplan–Meier survival curves for PFS in ETS and non-ETS patients in the entire population **b** Kaplan–Meier curves for OS in ETS and non-ETS patients in the entire population

Amongst all the patients, patients achieving ETS (n = 21) exhibited an improved PFS (*n* = 63; 6.5 vs 2.2 months, HR 0.523, *p* < 0.001) and OS (12.1 vs 6.0 months, HR 0.699, *p* = 0.014) compared with the non- ETS patients (Table 3, Fig. 2).

**Fig. 3:**
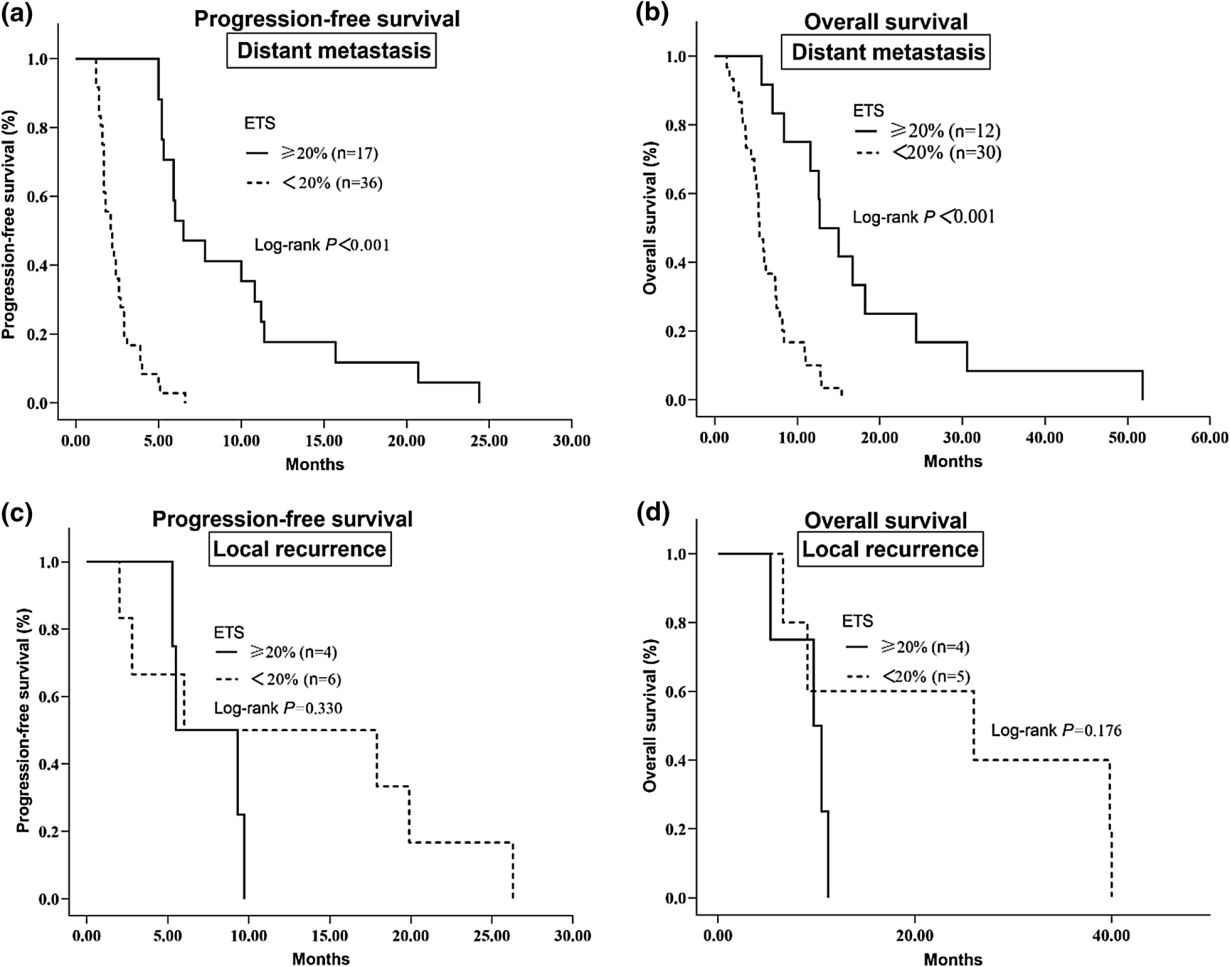
**a** Kaplan–Meier survival curves for PFS in ETS and non-ETS patients in patients with distant metastasis **b** Kaplan–Meier curves for OS in ETS and non-ETS patients in patients with distant metastasis **c** Kaplan–Meier curves for PFS in ETS and non-ETS patients in patients with local recurrence **d** Kaplan–Meier curves for OS in ETS and non-ETS patients in patients with local recurrence

Among the patients evaluated for ETS, 53 patients had distant metastasis, of which 17 (32.1%) achieved an ETS; while 10 patients had local recurrence, of which 4 reached an ETS. For the stratification analysis, among patients with distant metastasis, evaluation of PFS (6.5 vs. 2.1 months, HR 0.248, *p* < 0.001) and OS (13.9 vs. 5.4 months, HR 0.315, *p* < 0.001) favored ETS over non-ETS patients (Fig. 3). However, patients with local recurrence showed no significant association between ETS with both PFS (*p* = 0.330) and OS (*p* = 0.176) (Fig. 3).

### DpR and its stratification analysis related to survival

**Fig. 4:**
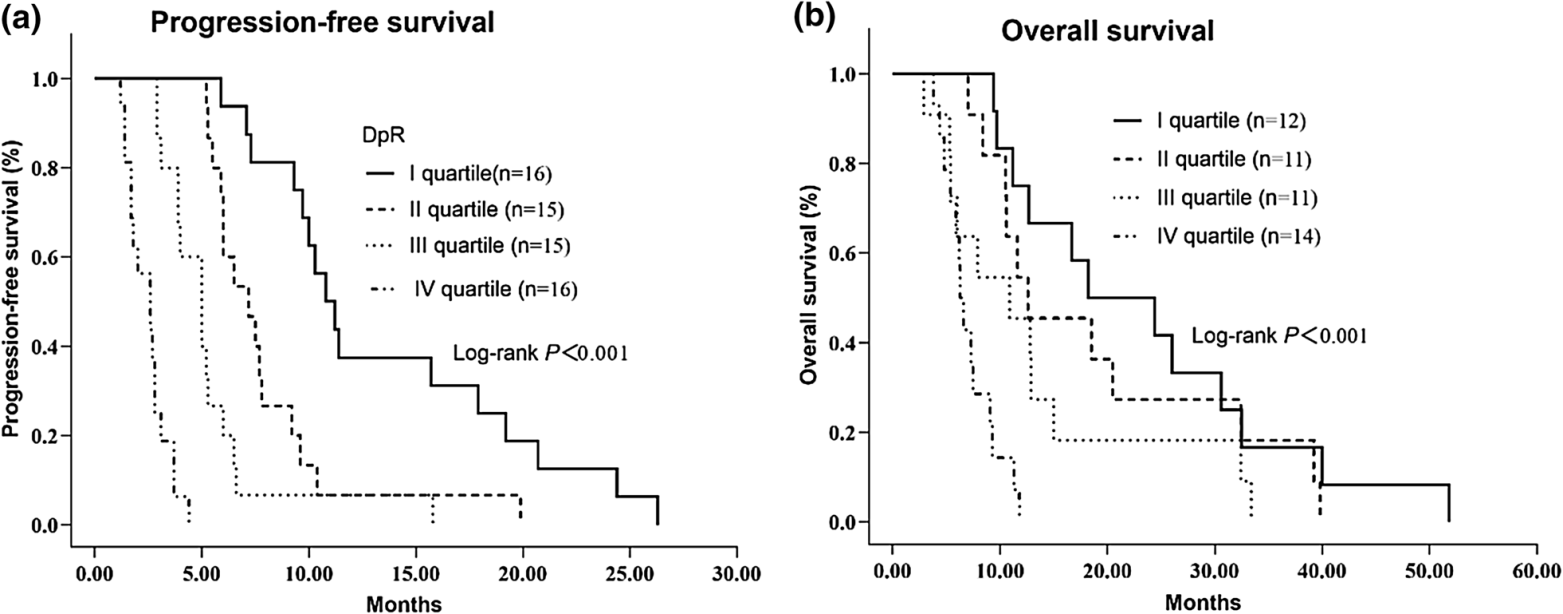
**a** Kaplan–Meier survival curves for PFS according to DpR quartiles in the entire population **b** Kaplan–Meier curves for OS according to DpR quartiles in the entire population

Amongst the study participants who were assessed, DpR, both as a discrete and a continuous variable, showed a strong link to both OS and PFS (Table 3, Fig. 4).

**Fig. 5:**
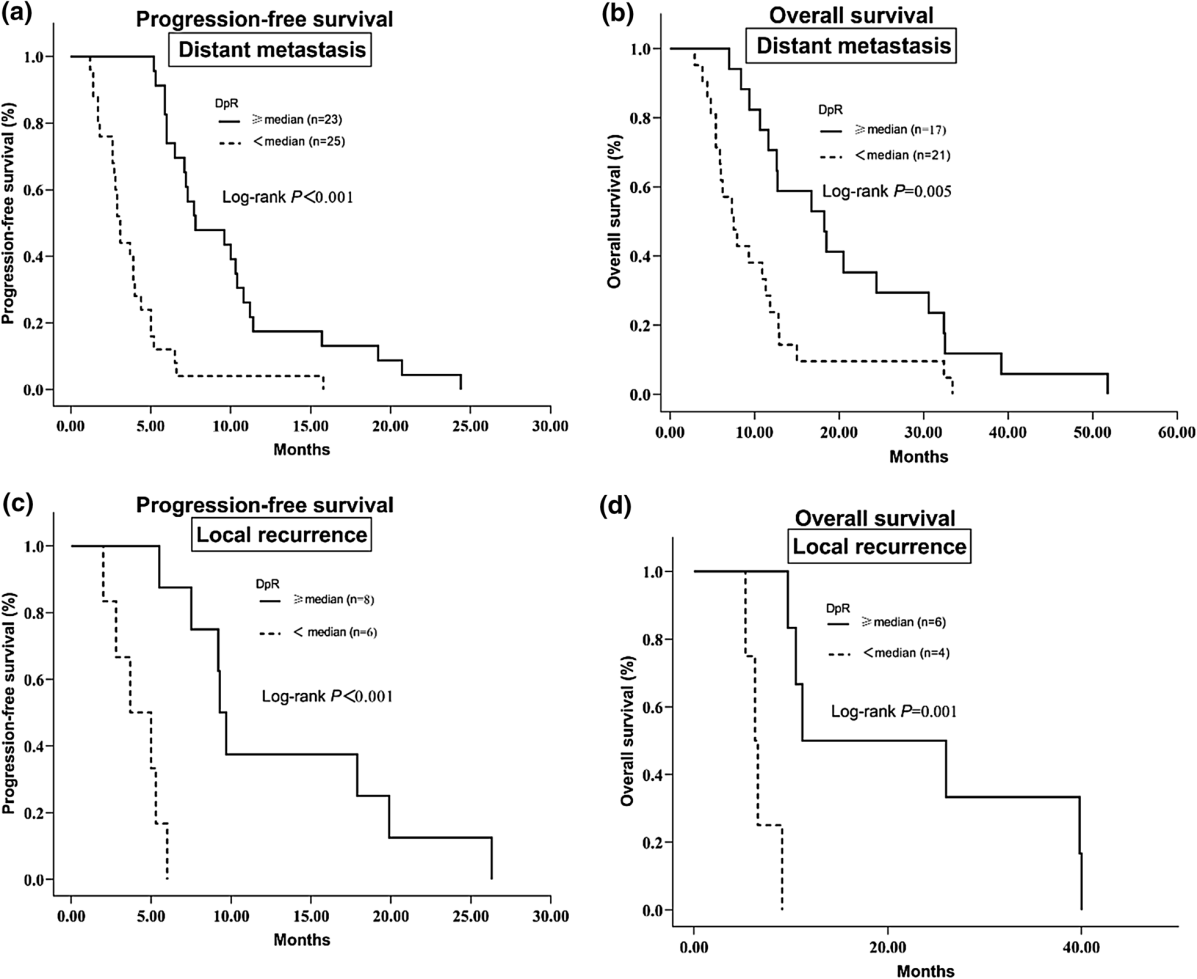
**a** Kaplan–Meier survival curves for PFS according to DpR median value among patients with distant metastasis **b** Kaplan–Meier curves for OS according to DpR median value among patients with distant metastasis **c** Kaplan–Meier curves for PFS according to DpR median value among patients with local recurrence **d** Kaplan–Meier curves for OS according to DpR median value among patients with local recurrence

Among the patients evaluated for DpR, 48 had distant metastasis, while the remaining 14 had local recurrence. For the stratification analysis, both in patients with local recurrence and distant metastasis, a deeper response was significantly linked to PFS and OS (*p* < 0.05) (Fig. 5).

### Prognostic factors multivariate analyses

The multivariable analysis performed using the Cox proportional hazards regression Model revealed stronger associations between both DpR as a continuous variable (*p* < 0.001) with a better PFS and ECOG PS 2 (*p* = 0.041) with a shorter PFS after adjusting for potential confounding variables. For OS, DpR (*p* < 0.001) and ETS (*p* = 0.001) both were determined to be independent prognostic factors. Furthermore, total number of metastatic sites ≥ 3 was associated with a poor prognosis for OS (*p* = 0.003) (Table 4).

**Table 4:** Multivariate analyses

Variables	PFS		OS	
	HR (95%CI)	*P-*value	HR (95%CI)	*P-*value
ECOG performance status (2)	4.682 (1.065–20.578)	**0.041**	–	–
Number of metastatic sites (≥ 3)	–	–	6.129 (1.840–20.418)	**0.003**
ETS (< 20%)	–	–	4.490 (1.842–10.946)	**0.001**
DpR (as a continuous variable)	1.027 (1.014–1.040)	** < 0.001**	1.037 (1.024–1.051)	** < 0.001**

Bold values indicate statistical significance at* P* < 0.05

*DpR* depth of response, *ECOG* Eastern Cooperative Oncology Group, *ETS* early tumour shrinkage

## Discussion

This study is the first report to examine the prognostic potential of ETS and DpR in first-line therapy for patients with RMPC, irrespective of the treatment regimens. The results of this analysis demonstrated that both ETS and DpR were significant independent predictors of a longer OS and were not dependent on the first-line treatment received. Moreover, while we observed a strong relationship between ETS and PFS/OS in patients with distant metastasis, a similar level of significance was not reached in patients with local recurrence; thus, suggesting that achieving an ETS > 20% might not be a long-term outcome predictor for patients with local recurrence.

In this study, we set the cut-off value at 20% for the optimal distinction between ETS and non-ETS, based on previous reports with advanced PC [14, 15]. The present study showed that 33.3% of the patients experienced ETS, which was consistent with the results of Kaga et al. (25.5%) [14] and Vivaldi et al. (35.5%) [15]. The median DpR was − 23.66%, which was also consistent with the previously reported percentages in the first published report to establish the prognostic value of DpR in PC[15].

However, previous two studies reported some conflicts [14, 15]. Kaga et al. reported that the ETS was not a independent predictor of OS [14]. Caterina et al. showed that DpR and ETS were significant associations with OS and PFS in one cohort; however, no statistical correlation was observed in the other cohort [15]. Our study indicated that both ETS and DpR were significant independent predictors of a longer OS. On conducting a multivariate regression analysis, including the metastatic sites, one of the available surrogate measures of tumor burden [10], DpR constituted an independent positive predictive factor of PFS and OS, but ETS was only maintained in our model involving OS. This suggested that the impact of ETS (versus non-ETS) was considerably higher on OS compared with PFS, consistent with the results for metastatic colorectal cancer from a review conducted by Heinemann et al. [18]. In the univariate analyses, ETS was not only associated with OS but also with PFS. One possible reason was that apart from a more favorable prognosis, ETS also distinguished patients who were highly sensitive to treatment from a heterogeneous group and identified patients who were early responders, which was also true for metastatic colorectal cancer [8, 18–21]. Therefore, in clinical trials, in order to prompt drug development and potentially orientate treatment decisions, such an endpoint would be extremely appealing. In routine practice, ETS ≥ 20% as a simple, reproducible parameter may predict outcomes and show the advantage of earlier assessment compared to RECIST response. Additionally, recent reports on mCRC claimed that presence of rapid and deep tumor shrinkage were linked with clear benefits in terms of rapidly relieving tumour-related symptoms, improving quality of life (QOL), delaying tumor progression and predictors of proceeding to conversion surgery [12, 22]. However, it is unclear whether these potential values exist in pancreatic cancer, thus deserving further investigations. Furthermore, it is important that further studies on PC include non-ETS as a heterogeneous group.

The stratification analysis of patients with distant metastasis or local recurrence revealed that the prognostic potential of ETS was retained in patients with distant metastasis, while patients with local recurrence lacked any substantial link with survival (PFS and OS). This suggested that the cut-off values (20%) set for the optimal distinction of patients with a more favorable prognosis among patients with local recurrence was not reasonable. Hubert et al. introduced the idea that further experiments may improve the prognostic potential of ETS by distinguishing between lymph nodes and organ metastases [10]. This study is the first report to investigate this parameter. However, since there were only a few patients with local recurrence (including regional lymph node metastases), additional research is necessary to verify these findings.

This study had several limitations. This is a retrospective single-center study that enrolled a limited number of patients. However, the results were consistent with previous reports that enrolled large populations. Additionally, the investigators performed the evaluations rather than by centralized radiological review, which might have introduced bias. Therefore, further large-scale prospective studies with international validation, ideally with centralized radiological assessment, is required.

## Conclusions

This study indicated that an earlier and deeper tumor shrinkage could anticipate the survival of advanced PC patients. These findings need further validation before using ETS and DpR in routine procedures of patient management.

## Supplementary Information

**Additional file 1: Table S1**: First-line treatment regimes.

## Data Availability

The datasets generated and/or analyzed in the present study are available from the corresponding author on reasonable request.

## Abbreviations

ETS: Early tumor shrinkage
DpR: Depth of response
PC: Pancreatic cancer
RMPC: Recurrent and metastatic pancreatic cancer
SLD: Sum-of-the-longest-diameters
PFS: Progression-free survival
OS: Overall survival
PPS: Post-progression survival
FOLFIRINOX: 5-Fluorouracil, irinotecan, and oxaliplatin
FOLFOXIRI: 5-Fluorouracil, irinotecan, and oxaliplatin
ECOG: Eastern cooperative oncology group
PS: Performance status
RECIST: Response evaluation criteria in solid tumors
CT: Computed tomography
HRs: Hazard ratios
CIs: Confidence intervals
GS: Gemcitabine plus S-1
GEMOX: Gemcitabine and oxaliplatin
DCR: Disease control rate
ORR: Objective response rate
