# Supplementary material for: Assessing prognostic value of early tumor shrinkage and depth of response in first-line therapy for patients with advanced unresectable pancreatic cancer
Source: BMC Gastroenterol. 2021 Jul 15;21:294. doi: 10.1186/s12876-021-01870-x (PMC8281486; doi:10.1186/s12876-021-01870-x)
Supplement: Supplementary file 1 — Additional file 1: Table S1: First-line treatment regimes. [file 12876_2021_1870_MOESM1_ESM.doc]

Table S1

first-line treatment regimes.

| Therapeutical regimes | Patients  *n*=89 | |
| --- | --- | --- |
|  | *n* | % |
| GS | 41 | 46.1 |
| FOLFIRINOX | 14 | 15.7 |
| GEMOX | 8 | 9.0 |
| G+Nab-paclitaxel | 6 | 6.7 |
| G+erlotinib | 4 | 4.5 |
| G | 4 | 4.5 |
| FOLFOXIRI | 2 | 2.2 |
| CPT-11+S-1 | 2 | 2.2 |
| Nab-paclitaxel+P | 2 | 2.2 |
| G+capecitabine | 1 | 1.1 |
| FOLFOX4 | 1 | 1.1 |
| XELOX | 1 | 1.1 |
| 5-FU+Nab-paclitaxel | 1 | 1.1 |
| GP | 1 | 1.1 |
| S-1 | 1 | 1.1 |
| 5-FU = 5-fluorouracil; CPT-11+S-1 = irinotecan plus S-1；FOLFIRINOX = 5-fluorouracil/irinotecan/ leucovorin/ oxaliplatin; FOLFOX4 = 5-fluorouracil/leucovorin/ oxaliplatin；FOLFOXIRI = 5-fluorouracil/irinotecan/ leucovorin/ oxaliplatin; GEMOX = Gemcitabine plus Oxaliplatin；G = gemcitabine；GP = gemcitabine plus cisplatin；  GS = gemcitabine plus S-1；XELOX = oxaliplatin plus capecitabine； | | |
